# Compensatory Evolution of Gene Regulation in Response to Stress by *Escherichia coli* Lacking RpoS

**DOI:** 10.1371/journal.pgen.1000671

**Published:** 2009-10-02

**Authors:** Daniel M. Stoebel, Karsten Hokamp, Michael S. Last, Charles J. Dorman

**Affiliations:** 1 Department of Microbiology and Moyne Institute of Preventive Medicine, School of Genetics and Microbiology, Trinity College, Dublin, Ireland; 2 Smurfit Institute of Genetics, School of Genetics and Microbiology, Trinity College, Dublin, Ireland; 3 UC Toxic Substance Research and Teaching Program, University of California Davis, Davis, California, United States of America; University of Toronto, Canada

## Abstract

The RpoS sigma factor protein of *Escherichia coli* RNA polymerase is the master transcriptional regulator of physiological responses to a variety of stresses. This stress response comes at the expense of scavenging for scarce resources, causing a trade-off between stress tolerance and nutrient acquisition. This trade-off favors non-functional *rpoS* alleles in nutrient-poor environments. We used experimental evolution to explore how natural selection modifies the regulatory network of strains lacking RpoS when they evolve in an osmotically stressful environment. We found that strains lacking RpoS adapt less variably, in terms of both fitness increase and changes in patterns of transcription, than strains with functional RpoS. This phenotypic uniformity was caused by the same adaptive mutation in every independent population: the insertion of IS*10* into the promoter of the *otsBA* operon. OtsA and OtsB are required to synthesize the osmoprotectant trehalose, and transcription of *otsBA* requires RpoS in the wild-type genetic background. The evolved IS*10* insertion rewires expression of *otsBA* from RpoS-dependent to RpoS-independent, allowing for partial restoration of wild-type response to osmotic stress. Our results show that the regulatory networks of bacteria can evolve new structures in ways that are both rapid and repeatable.

## Author Summary

*Escherichia coli*, like all bacteria, expresses distinct sets of genes in response to different environmental challenges. One protein, RpoS, is a central part of the cellular response that brings about these changes in gene expression. Despite the importance of this protein in response to some kinds of stresses, strains that lack a functional RpoS protein are found at appreciable frequency in nature. We sought to understand how these strains evolve to compensate for the lack of RpoS function. We evolved *E. coli* with and without RpoS in a stressful environment in the lab, and found that strains without RpoS evolved in a uniform and repeatable manner. This was true in terms of how much their fitness increased or in terms of how genes were expressed to compensate for the lack of RpoS. These patterns had a simple cause. A mobile genetic element moved position in the genome, allowing for the transcription of a pair of key genes. The same element moved to the same place in each of our replicate experiments, causing the repeatable change in fitness and gene expression. We conclude that *E. coli* can rapidly compensate for the lack of RpoS by evolving novel mechanisms to control patterns of gene expression.

## Introduction

Bacterial adaptation to environmental stress involves, in part, a modification of transcription patterns, with downstream impacts on physiological function. In *Escherichia coli*, the RNA polymerase sigma factor RpoS is a global regulator that coordinates the expression of up to 10% of the genome when the bacterium enters stationary phase or experiences stresses such as starvation, acidity or increased osmolarity [1]. Despite the importance of this protein in many environments, a functional RpoS seems to lower the ability of *E. coli* to scavenge for scarce nutrients [2],[3]. This cost is hypothesized to occur because there is a limiting amount of core RNA polymerase subunits in the cell, meaning that transcription of stress responsive, RpoS-dependent promoters will decrease the transcription from RpoS-independent promoters involved in nutrient acquisition and utilization [2]–[4].

The hypothesis that the nature of the RpoS regulatory network creates an inherent conflict between stress protection and nutritional competence (SPANC) [3],[4] provides a basis for predicting how natural selection acts on the global regulatory networks of *E. coli*. The SPANC hypothesis predicts that natural selection will modify the network in favor of nutritional ability at the expense of stress resistance in some environments by decreasing or abolishing RpoS function. Just this type of selection against RpoS activity has been documented in laboratory studies [2],[5],[6]. In addition, strains with low- or null-activity *rpoS* alleles have been found in natural populations of *E. coli* and *Salmonella enterica* [3],[7].

While strains without functional RpoS are favored in some environments, those same strains may do less well in other, more stressful environments where they may be less fit due to an inability to respond to new challenges. While *rpoS* strains could adapt by recovering or increasing their RpoS function, the mutations that abolished RpoS function may be very unlikely or impossible to reverse. An alternative mechanism involves the selection of mutations that modify the regulatory network to compensate for the loss of RpoS. These compensatory mutations would then increase the fitness of the bacterium in this new, more stressful environment. We sought to understand this type of adaptation by observing the patterns of increased fitness seen in evolving bacterial lines, and by elucidating the molecular basis of the adaptation.

Results from previous experimental studies suggest that compensation for deleterious mutations is a general phenomenon [8]–[11]. Less is known, however, about the variability of the process of compensation. Will strains that lack RpoS adapt more or less variably to a stressful environment than strains with a fully functional regulatory network? Will this involve larger or smaller increases in fitness? At a molecular level, mutations affecting other global regulators of transcription [1], or local changes at a promoter [12],[13] may permit transcription in the absence of RpoS, but we were interested in discovering which options actually are favored by natural selection. Would only a few key genes be involved in adaptation, or would adaptation involve changes in large parts of the transcriptome? Here we used experimental evolution [14],[15] to answer these questions about adaptation to the loss of RpoS.

## Results

*E. coli* expresses RpoS in response to stresses such as extremes of pH, temperature or osmolarity. If this regulatory pattern is important for fitness, then strains lacking RpoS should be less fit in these stressful environments. To test this hypothesis, we carried out a competition in a high osmolarity environment between a strain with wild-type *rpoS* and a strain with a deletion of the *rpoS* locus. As expected, the Δ*rpoS* strain was less fit than its wild-type ancestor (Table 1). This was not due to another mutation elsewhere in the genome, as other reconstructions of this same strain pairing showed the same cost (i.e. the difference in fitness) (Table 1, ANOVA, p = 0.89). The fitness cost was only slightly larger when the bacteria were grown with 0.44 M sucrose (t-test, p = 0.01), indicating that there was a general cost of osmotic stress and not only a salt-specific cost. Furthermore, the cost was specific to high osmolarity, as there was no fitness cost when the two stains were competed in the absence of NaCl stress (Table 1). Finally, the fitness cost was not due the activity of the kanamycin resistance gene used to knock out *rpoS*, as the same resistance cassette placed at the *melB* locus had no fitness cost (Table 1).

**Table 1:** Fitness results.

Experiment	Culture conditions	Mean fitness difference±standard error of the mean	Sample size
Δ*rpoS* vs *rpoS^+^* (DMS1688 vs DMS1684)	MOPS MM+0.3 M NaCl	0.83±0.04^a^	4
Δ*rpoS* vs *rpoS^+^* (DMS1717 vs DMS1711)	MOPS MM+0.3 M NaCl	0.84±0.01^a^	4
Δ*rpoS* vs *rpoS^+^* (DMS1727 vs DMS1726)	MOPS MM+0.3 M NaCl	0.82±0.01^a^	4
Δ*rpoS* vs *rpoS^+^* (DMS1688 vs DMS1684)	MOPS MM+0.44 M sucrose	0.78±0.01^a^	4
Δ*rpoS* vs *rpoS^+^* (DMS1688 vs DMS1684)	MOPS MM	0.97±0.04^b^	5
Wild type vs. *melB::kan* (DMS1692 vs DMS1766)	MOPS MM+0.3 M NaCl	1.00±0.03^b^	4

a value significantly different from 1, p<10^−5^, t-test.

b value not different from 1, p>0.3, t-test.

**Figure 1:**
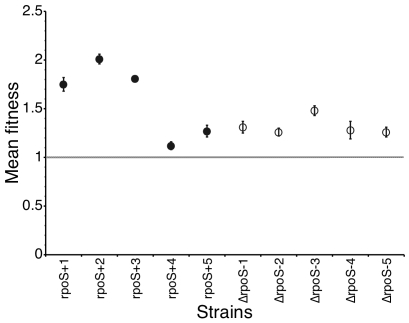
Fitness of evolved strains compared with their ancestor. Five *rpoS^+^* lines (filled circles) were more variable in their fitness increase than the five Δ*rpoS* lines (open circles). The dashed line shows fitness of 1, indicating equal fitness between two competitors. Each competition experiment was replicated four times, and error bars represent the standard error of the mean.

To allow strains to adapt to this high-osmolarity environment, we serially cultured five *rpoS* ^+^ lines (denoted *rpoS*+1 to *rpoS*+5) and five Δ*rpoS* lines (Δ*rpoS*−1 to Δ*rpoS*−5) in a high-osmolarity medium for 250 generations, and isolated a single colony from the final population. When competed against their ancestor, all 10 evolved lines showed an increase in fitness (Figure 1). This was due, at least in part, to the fact that each increased its growth rate (Table S1). Importantly, and unexpectedly, there was no significant difference in the average fitness increase of the Δ*rpoS* lines and the *rpoS^+^* lines (t-test, p = 0.18). In addition, the variance in fitness among the Δ*rpoS* lines was smaller than the variance among the *rpoS* ^+^ lines (F-test, p = 0.02).

**Figure 2:**
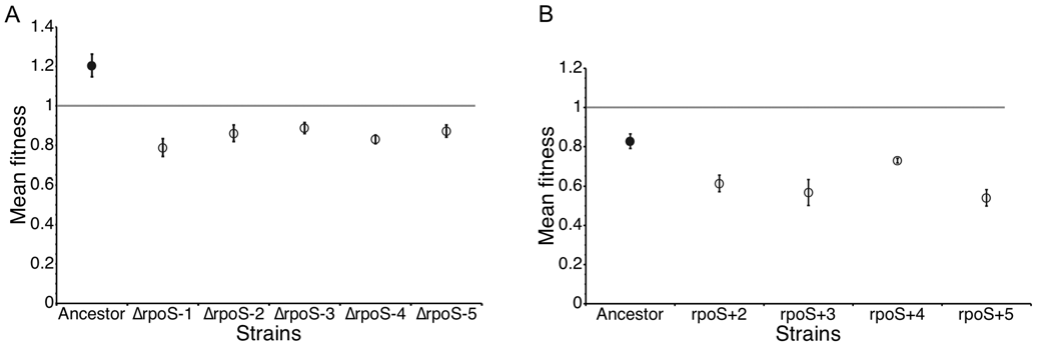
Adaptation was contingent on the status of *rpoS*. A functional *rpoS* allele was transferred into each of the five Δ*rpoS* lines, and each of Δ*rpoS* strains was competed against this newly *rpoS^+^* strain (A). In the wild-type background, strains with *rpoS^+^* are more fit than Δ*rpoS* (filled circle), indicated by fitness greater than 1. All five evolved backgrounds are *less* fit as *rpoS^+^* than Δ*rpoS* (open circles), as indicated by fitness less than 1. The dashed line shows fitness of 1, indicating equal fitness between the two competitors. A Δ*rpoS* line was derived from the *rpoS^+^* lines, and each of these pairs of strains was competed (B). While Δ*rpoS* is less fit on the wild-type background (filled circle), Δ*rpoS* is even more detrimental for lines *rpoS*+2, *rpoS*+3, and *rpoS*+5 (open circles). Line *rpoS*+1 evolved resistance to P1, so the transduction could not be performed.

Was the fitness increase of the Δ*rpoS* lines a compensation for the lack of RpoS function, or simply a general adaptation to this environment? If it was compensation for lack of RpoS function, then adaptation should be epistatic on *rpoS*. To test this possibility, we restored the Δ*rpoS* lines to *rpoS^+^*. While *rpoS^+^* is favored over Δ*rpoS* in the ancestral genetic background, all five Δ*rpoS* lines became less fit when transduced from Δ*rpoS* to *rpoS^+^* (Figure 2A), indicating that fitness increased by compensating for the lack of RpoS, rather than by general adaptation to the culture conditions. To see if the *rpoS* ^+^ lines adapted in an RpoS-dependent manner, we transduced these lines from *rpoS^+^* to Δ*rpoS*. Although Δ*rpoS* was costly in the ancestral genetic background, it was even more costly in three of four of the evolved *rpoS* ^+^ genetic backgrounds (p<0.05) (Figure 2B), indicating that adaptation of these lines was via an RpoS-dependent mechanism.

We used DNA microarrays to explore the patterns of regulatory evolution underlying our observed fitness changes. To assess if our observed patterns of mean change and variance in fitness were mirrored by changes in the transcriptome, we compared the expression pattern of both the *rpoS* ^+^ and Δ*rpoS* lines to that of their ancestors. For each of the 4,254 genes represented on the array, we calculated the difference of each of the 10 evolved lines from its ancestor. For each gene, we then asked whether the average difference was larger for the *rpoS* ^+^ or the Δ*rpoS* populations. The *rpoS* ^+^ lines were more different from their ancestor than the Δ*rpoS* lines were from their ancestor for 61% of genes, significantly more than would be expected by chance (p<10^−16^, sign test). We used the same approach to assess the variability in expression patterns, and found that the expression of 90.7% of genes was more variable in the *rpoS* ^+^ lines than in the Δ*rpoS* lines (p<10^−16^, sign test). The expression level of most genes was unchanged in most strains (Table 2), so we repeated this analysis with the expression level set to the ancestral value for all measurements that did not pass our statistical threshold (FDR = 0.001). Of those genes that showed change, 91.5% changed more in the *rpoS^+^* lines than in the Δ*rpoS* lines (p<10^−16^, sign test). Further, 92.3% of genes were more variable in the *rpoS^+^* lines than the Δ*rpoS* lines (p<10^−16^, sign test). These results neatly paralleled our fitness results, showing that highly similar trajectories of fitness increase in Δ*rpoS* were underlain by similarly parallel changes in patterns of transcription.

**Table 2:** Number of genes with differing levels of expression.

Comparison	Number of genes differentially expressed
*rpoS^+^* vs. Δ*rpoS*	331
*rpoS^+^* vs *rpoS*+1	1131
*rpoS^+^* vs *rpoS*+2	74
*rpoS^+^* vs *rpoS*+3	47
*rpoS^+^* vs *rpoS*+4	38
*rpoS^+^* vs *rpoS*+5	609
Δ*rpoS* vs Δ*rpoS*−1	83
Δ*rpoS* vs Δ*rpoS*−2	87
Δ*rpoS* vs Δ*rpoS*−3	156
Δ*rpoS* vs Δ*rpoS*−4	81
Δ*rpoS* vs Δ*rpoS*−5	95

**Figure 3:**
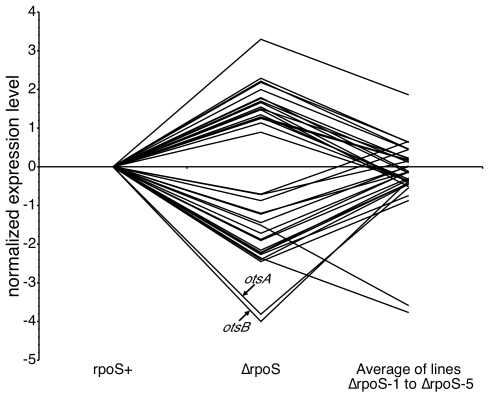
Expression of the 37 genes with changed expression patterns in all five Δ*rpoS* lines. For 35 of 37 genes, the mean level of expression in the evolved lines is closer to the *rpoS^+^* level than the Δ*rpoS* ancestor is. The log-transformed expression levels normalized to the *rpoS^+^* value are shown. *otsB* and *otsA* are the two genes with the lowest values in the Δ*rpoS* strain.

The fitness results also showed that the Δ*rpoS* lines evolved to compensate for the lack of RpoS function. Did they do so by returning transcription back towards original wild-type levels, or did adaptation result in the transcriptome becoming even more different from wild type? We found that 331 genes (or 7.8% of the genome) differed significantly in expression between the ancestral wild type and the ancestral Δ*rpoS* strains during growth in high osmolarity media. The evolved Δ*rpoS* lines showed significant changes in the level of expression of between 81 and 156 genes from the Δ*rpoS* ancestor (Table 2). Of these, 37 were changed significantly in all five lines (Table S2). All 37 were genes that also differed between wild type and Δ*rpoS*. We compared the average expression level of these 37 genes in the five Δ*rpoS* lines with their level in their Δ*rpoS* ancestor, and in the *rpoS^+^* progenitor. In 35 of 37 cases, the expression level of the Δ*rpoS* lines evolved to be more similar to *rpoS^+^* than was their Δ*rpoS* ancestor (Figure 3). Thus, compensation for the lack of RpoS function involved partial restoration of the wild-type pattern of transcription.

What sorts of mutations brought about these parallel changes in the expression of 37 genes? To address this question, we focused first on the two genes with the most dramatic changes in expression in the evolved lines. These genes, *otsB* and *otsA* were upregulated an average of 12.5 and 9.7 fold, respectively, in the Δ*rpoS* lines. These genes code for the two enzymes required for trehalose biosynthesis and *E. coli* synthesizes trehalose under osmotic stress in order to achieve internal osmotic balance [16]. *otsB* and *otsA* form an operon (*otsBA*) that requires RpoS for transcription in wild-type *E. coli* [17], suggesting that the Δ*rpoS* lines had evolved RpoS-independent expression of this operon.

To determine whether this new expression pattern was via a mutation in the *otsBA* promoter or a mutation elsewhere in the genome, we sequenced the *otsBA* promoter of all five lines and found that all five contained identical IS*10* insertions. This insertion was located between the +12 and +13 bases relative to the transcriptional start site determined by Becker and Hengge-Aronis [18]. IS*10* contains a promoter, P_OUT_, directed outward from IS*10* into adjacent DNA [19],[20]. In all five Δ*rpoS* lines, IS*10* was oriented with P_OUT_ reading into the *otsB* gene. IS*10* is known to have strong sequence preferences for insertion, and while the site of this insertion at *otsB* resembles the preferred site, it is not optimal. There is a marked preference for the symmetric site 5′-GCTNAGC-3′, but we found insertion at 5′-GTAAAGC-3′. The presence of a thymine instead of a cytosine at the second position lowers the insertion frequency over 1,000 fold from the preferred site in another tested context [21].

Surprised to find the same mutation in all five lines, we wanted to eliminate the possibility that this mutation fixed in all five lines because it occurred once before the starting culture was split into the five separate lines. If the mutation occurred in all five lines independently, i.e. after the culture was split, then we reasoned that if we started five completely independent lines, each should acquire the same IS*10* insertion into P*_otsBA_*. To test this hypothesis, we spread an aliquot of the original Δ*rpoS* frozen culture onto an L agar plate, picked five separate colonies (thus each founded by a single cell) and used each of these to found a new long-term line. Diagnostic PCR of the P*_otsBA_* promoter confirmed that each of these lines began with a wild-type promoter. These lines were evolved for 250 generations under identical conditions to those used in the first experiment. After 250 generations, all five of these lines had acquired an IS*10* insertion mutation in the same location and with the same orientation as the first five lines, indicating that the repeated evolution of this particular promoter mutation is not due to the mutation having been present in the starting population.

While all five Δ*rpoS* lines fixed the same mutation, the dynamics of this mutational sweep need not have been uniform. To explore this, we used QPCR to determine the frequency of the IS*10* insertion into P*_otsBA_* after 80 generations. The frequency of the insertion varied over two orders of magnitude (Table S3), and the frequency in all five cultures was distinct. This could be due to the insertion occurring at distinct time points in each line, or due to the initial rise of the adaptive mutation being dominated by the stochastic dynamics of culture transfer from flask to flask.

**Figure 4:**
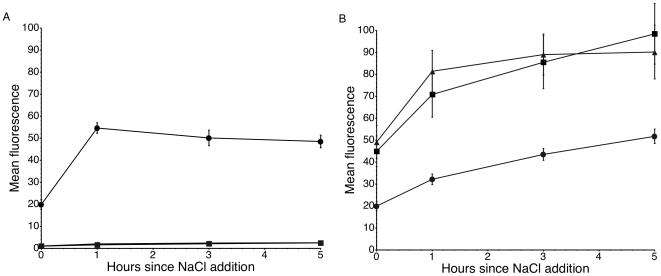
Expression of P*_otsBA_* measured by a *gfp* transcriptional fusion. Cells were transformed with a plasmid expressing *gfp* either from the wild-type P*_otsBA_* (A) or from P*_otsBA_* with IS*10* inserted (B) into one of three genetic backgrounds: *rpoS^+^* (circles), Δ*rpoS* (squares) or Δ*rpoS*−1 (triangles). Cells were grown over-night in MOPS MM, then diluted 1∶100 into fresh media and grown until they reached an OD_600_ of 0.25. At this point (time 0) a sample was taken, and NaCl was added to a final concentration of 0.3 M. Fluorescence was measured on a flow cytometer. The experiment was repeated three times, and error bars represent the standard error of the mean.

To determine if the IS*10* insertion alone was sufficient to allow for RpoS-independent expression of *otsBA*, we cloned the wild-type and evolved P*_otsBA_* promoters into the promoterless *gfp*-fusion plasmid pZep08 [22]. In the *rpoS^+^* ancestor, the wild-type promoter was expressed even in low-osmolarity MOPS minimal medium, and was upregulated upon the addition of NaCl (Figure 4A). The P*_otsBA_* promoter remained un-expressed in both the ancestral Δ*rpoS* background, and in the Δ*rpoS*−1 line. On the other hand, the evolved (IS*10* inserted) P*_otsBA_* promoter was expressed in all three strains (Figure 4B). The ancestral Δ*rpoS* line and the Δ*rpoS*−1 line expressed at similar levels, suggesting that there was not a second mutation beyond the IS*10* insertion that allowed for *otsBA* expression in the evolved lines. The evolved promoter was expressed at a lower level in the *rpoS^+^* strain than in either Δ*rpoS* line, which may explain why the evolved lines became less fit when made *rpoS^+^*.

**Figure 5:**
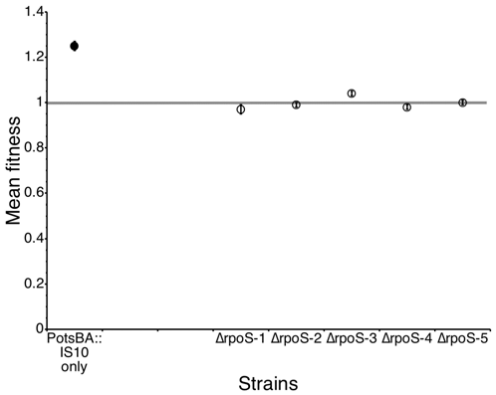
Fitness effects of the P*_otsBA_*::IS*10* insertion. The fitness of a Δ*rpoS::kan* strain with the P*_otsBA_*::IS*10* insertion introduced (filled circle) has fitness of 1.25 relative to the Δ*rpoS::kan*, a value equivalent to the evolved lines. Four of the five evolved lines, when transduced to wild type P*_otsBA_* (open circles), have fitness no different from Δ*rpoS::kan*. The dashed line shows fitness of 1, indicating equal fitness between the two competitors.

What is the fitness effect of this IS*10* insertion? When the IS*10* insertion was moved into the ancestral Δ*rpoS* background, we found that the newly constructed strain had a fitness of 1.25 when competed against the Δ*rpoS* strain. The fitness advantage due solely to the IS*10* insertion was not different from the fitness of the five evolved Δ*rpoS* lines (ANOVA, p>0.05), suggesting that the IS*10* insertion was responsible for all of the adaptation. To complement these experiments, we transduced all five of the Δ*rpoS* evolved lines to a wild-type *otsBA* promoter. These strains were then competed against their Δ*rpoS* ancestor, and four of the five were now found not to be different to their ancestor (Figure 5). Only the Δ*rpoS*−3 line was significantly fitter than its ancestor when transduced to wild-type *otsBA* (fitness = 1.04, p = 0.003), suggesting that it has a second mutation beyond the IS*10* insertion at that locus. There is no evidence that the other four strains have any other mutation that affects fitness in the high-osmolarity environment.

**Figure 6:**
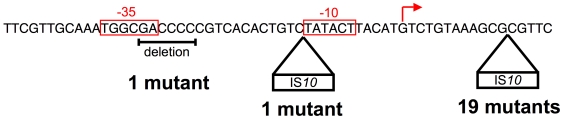
The position of 21 mutants that upregulate an *otsB::lacZY* fusion. The 19 IS*10* insertions between +12 and +13 are in the same position as those recovered from the experimental evolution, and all 19 mutants have IS*10* inserted with P_OUT_ oriented reading into *otsB*. Likewise, the IS*10* inserted between −11 and −12 has P_OUT_ oriented reading into *otsB*. The start of translation is at +56. The transcriptional start site is from [18], and the −10 and −35 sites are inferred from the data in [23].

Did all five of the lines that contained the same IS*10* do so because this was the only way to upregulate *otsBA*, or do other routes exist? To answer this question, we selected mutants that could upregulate an *otsB-lacZY* transcriptional fusion in a Δ*rpoS* background. Of 21 independent mutants, 19 possessed the same IS*10* insertion as we recovered from our experimental evolution, while another had an IS*10* insertion between bases −11 and −12 in the promoter (Figure 6). Finally, one mutant had a 6-bp deletion overlapping the −35 box of the promoter [18]. While the wild-type −35 (TGGCGA) box of the P*_otsBA_* promoter differs strongly from the RpoD −35 consensus (TTGACA) [23], a sequence closer to the consensus (TTGCAA) lies just upstream in the wild-type promoter. The 6-bp deletion moved this other sequence into position to serve as a −35, presumably allowing RpoD-dependent transcription. These results demonstrate that other mutational routes to upregulation exist, but the IS*10* insertion observed in our experimental evolution is the most likely to occur.

## Discussion

The nature of the regulatory network in *E. coli* that governs its response to stress creates trade-offs between fitness in high and low-stress environments. If selection cannot increase the RpoS-dependent response to stress, then novel regulatory mechanisms may evolve to compensate. We have experimentally evolved both *rpoS^+^* and Δ*rpoS* lines in an osmotically stressful environment and explored how these populations adapted to this stress. We were surprised to find that the Δ*rpoS* populations evolved via the same mutation in each line, which did not result in a larger increase in fitness than the *rpoS^+^* lines. We had expected that because they were less fit, the Δ*rpoS* lines would fix mutations causing a larger average fitness increase, as has been found in other cases of experimental evolution for compensation of deleterious mutations [9],[24]. The expectation of larger fitness increases is supported by models of the genetics of adaptation that follow from Fisher [25],[26]. These models also predict that less fit genotypes will have more possible mutations resulting in more variation in fitness increases. We found the opposite: there was *less* variation in the fitness increase of Δ*rpoS* lines, due to the fact that the same mutation fixed in all five populations.

One possible explanation for our observation is that the deletion of *rpoS* causes the strain to cross a fitness valley, and places it on a smaller peak. On this new, smaller peak, adaptive mutations would be of smaller size, and there would be fewer of them, resulting in more parallel evolution. In biological terms, this implies that it is possible that trehalose biosynthesis is so critical that mutations upregulating *otsBA* will be much more strongly favored than any other adaptive mutation. If this is the case, then recovery of the same mutation in replicate lines may reflect the limited number of possible adaptive mutations that can upregulate *otsBA*. Alternatively, it may reflect the fact that the site in the promoter is a hotspot for IS*10* insertion. This latter possibility is supported by our observation that 90% of the mutants up-regulating *otsBA* that we recovered in our screen contained the same IS*10* insertion as found in our experimental evolution.

All of the evolution in our Δ*rpoS* lines was mediated by IS*10*. While this constancy may be unusual, IS elements have been frequently found as the causes of adaptive mutations in experimental evolution [27]–[29]. In addition, comparative genomics suggests that IS elements play an important role in genome evolution [30]–[32]. While the specific role of IS elements in regulatory evolution has been less thoroughly explored, a wide range of IS elements are known to activate transcription by insertion into promoters [12]. Further, IS elements are found in almost all strains of *E. coli*. In their survey of a representative collection of *E. coli* isolates, Sawyer *et al.* [33] found that 97% contained at least one of the six IS elements for which they screened. They did not screen for IS*10*, but genomic DNA sequencing reveals that it is not rare: it is present in 16% of *E. coli* and *Shigella* genomes in GenBank. Thus, it is not improbable that IS elements play an important role in the evolution of the RpoS regulon.

The IS*10* insertion in P*_otsBA_* affected the transcription of a number of genes beyond only *otsBA*. The process of compensation for a fitness cost might result in two sorts of patterns of change of transcription. In the first, deletion of RpoS changes the level of transcription of a gene, and compensatory adaptation brings the level of transcription back towards the wild-type level. An alternative is that the process of compensation results in more change in the level of transcription. For example, a gene that is somewhat down-regulated with the loss of RpoS becomes even further down-regulated, or a gene that was not initially affected by the loss of RpoS is upregulated to compensate for the lack of transcription of some other gene. We found that of the 35 genes other than *otsBA* with changed pattern of transcription across all 5 lines, 33 fell into the former category of (partial) restoration of transcription levels. OtsB and OtsA are biosynthetic enzymes, not transcription factors, but the small molecule they synthesize, trehalose, does stimulate expression of genes involved in maltose transport [34], and the *malK-lamB-malM* operon is up-regulated in the evolved lines. Other changes in transcription may be responses to other restored physiological states, such as growth rate (the gene *rmf* [35]), or osmotic balance (*proP* [36], *pspABC* [37]). A general implication of these observations is that regulatory systems may be structured such that in the absence of reversion at the genetic level, compensatory mutations can lead to restoration of wild-type patterns of transcription.

What are the consequences for future evolution of compensating for the lack of RpoS with an IS*10* insertion? The SPANC model put forth by Ferenci and co-workers [3],[4] proposes that selection will favor decreased levels of RpoS activity in nutrient-poor environments, and increased levels in stressful environments. If null *rpoS* alleles that cannot be reverted are fixed in nutrient-poor environments, then strains may compensate in stressful environments with mutations elsewhere in the regulatory network. Because epistasis is generated in this compensation, subsequent re-acquisition of RpoS function will decrease fitness. Strains that have taken the first step of adapting in an RpoS-independent manner will continue to do so, leading to even further divergence of regulatory networks.

A mechanism to explain the origins of the observed epistasis, sigma factor competition [38],[39], suggests that epistasis is likely to be a general phenomenon. Sigma factor competition is hypothesized to occur because amounts of core RNA polymerase are limiting for transcription. Thus, if an RpoS molecule interacts with a core subunit to promote transcription from one promoter, there is one less core subunit available for any other sigma factor to interact with and promote transcription. Since the P_OUT_ promoter of IS*10* is not an RpoS-dependent promoter, the presence of functional RpoS protein will reduce the levels of transcription from promoters like P_OUT_. Because this cause of epistasis is embedded in the fundamental process of transcription, it suggests that mutations recruiting non-RpoS-dependent promoters to compensate for the loss of RpoS will generally be epistatic on the absence of RpoS.

The RpoS regulatory network is a major target for selection because it cannot handle environments that are both physically stressful and nutrient-poor. The SPANC model posits that differing levels of RpoS activity will be selected in different environments. Our work has shown an alternative to increasing RpoS levels in a stressful environment. Strains adapt with a repeatable pattern by up-regulating a single pair of genes on the periphery of a regulatory network, suggesting that regulatory networks may evolve novel structures in a rapid and predictable manner.

## Materials and Methods

### Strains, plasmids, and media

All strains used are listed in Table S4. Text S1 notes the location of the two copies of IS*10* in the ancestral strain used for experimental evolution. Long-term evolution and competition experiments were conducted in MOPS minimal medium [40] with 0.2% glucose (hereafter MOPS MM) as a carbon source. In most experiments an additional 0.3 M NaCl added for osmotic stress. For some experiments no additional NaCl was added, or 0.44 M sucrose was used instead of NaCl. L medium was 0.5% yeast extract, 1% tryptone, 0.5% NaCl. Antibiotics were used at 15 mg l^−1^ tetracycline, 50 mg l^−1^ kanamycin, 100 mg ml^−1^ carbenicllin, and 20 mg ml^−1^ chloramphenicol.

### Long-term experimental evolution

Strains were grown in 25 ml of MOPS MM+0.3 M NaCl in 250 ml flasks, shaken at 200 rpm at 37°C. 25 µl of culture was transferred to 25 ml of fresh media every 24 hours. This 1∶1000 dilution results in log_2_(1000) = 9.96 doublings per day. The long-term experiment was conducted for 25 days, or approximately 250 generations. Cultures were frozen at −80°C by the addition of glycerol to 20%. At the start of the experiment, a single *rpoS^+^* colony (DMS1684) and a single Δ*rpoS* colony (DMS1688) were chosen from L agar plates and grown overnight in 1 ml MOPS MM. The next day, 250 µl of culture was added to 25 ml of MOPS MM and grown for two hours at 37°C, shaken at 200 rpm. NaCl was then added to a final concentration of 0.3 M, and cultures were grown for another 22 hours. The next day, these cultures were used to found the five *rpoS^+^* and five Δ*rpoS* lines by inoculating 25 µl into 25 ml fresh medium.

### Competition experiments

To compete a pair of strains, each was first inoculated directly from −80°C frozen culture into 1 ml of MOPS MM in a culture tube and grown overnight at 37°C. The next day, 250 µl of culture was added to 25 ml of MOPS MM and grown for two hours at 37°C, shaken at 200 rpm. NaCl or sucrose was added to a final concentration of 0.3 M or 0.44 M as appropriate to the experiment, and cultures were grown for another 22 hours. To initiate the competition, equal volumes of the two strains were mixed, and 25 µl of mixed culture was added to 25 ml of fresh MOPS MM (plus NaCl or sucrose as appropriate). Cells were grown for 24 hours.

Samples of cells at the start and end of the competition were diluted in MOPS MM without glucose or K_2_HPO_4_ and then plated on L agar and on a plate that distinguished the two strains. When the competitors differed by an antibiotic resistance marker, this antibiotic was used to distinguish them. If they did not differ in this way, a mutation in *fhuA*, conferring resistance to phage T5 was used [41]. This mutation has no effect on fitness (data not shown).

Fitness was calculated as the ratio of the growth rates of the two strains, as described [42]. Differences in plating efficiency between two strains do not effect the calculation of fitness [42].

### Strain construction

Alleles were moved between strains by P1 transduction [43]. Movement of the *rpoS^+^* allele into a Δ*rpoS* background was accomplished by co-transduction with a *tetRA* element inserted into the *ygbM* locus. *tetRA* was amplified from strain CAG18642 by primers *ygbM*_*tetRA*+ and *ygbM*_*tetRA*− (Table S5) and recombined into the *ygbM* locus via the lambda-red proteins expressed off plasmid pKD46 [44],[45]. The location of the insert was confirmed by sequencing with primers *ygbM*_sequence+ and *ygbM*_sequence−. Movement of wild-type and IS*10* inserted P*_otsBA_* alleles was via a linked *tetRA::araH* element. To move the wild-type promoter, *tetRA* amplified by primers *araH*_*tetRA*+ and *araH*_*tetRA*− and was inserted into *araH* of DMS1684 via pKD46 recombination as above. To move the P*_otsBA_*::IS*10* allele, *tetRA* was first inserted into *araH* of the evolved strain DMS1745. Then, the entire *tetRA* element and P*_otsBA_*::IS*10* was amplified by PCR using primers *araH*_*tetRA*+ and *otsB*_recomb− and recombined into DMS1684. The insert was confirmed by sequencing with primers *araH*_*tetRA* verify+ and *araH*_*tetRA* verify−.

Construction of a *otsB::lacZYcat* mutant was a two step process. First, the *cat* gene was PCR amplified from plasmid pKD3 [44] using primers *cat*_*lacA*+ and *cat*_*lacA*−, and recombined into the *lacA* gene of MG1655 via pKD46 mediated recombination to create strain DMS1976. *lacZYcat* was then PCR amplified with primers *otsB*_cds_*lacZ*_fusion+ and *otsA*_cds_*lacZ*_fusion−. These primers included an in-frame stop codon and amplified the native *lacZ* ribosome binding site. The PCR product was inserted into the *otsB* gene of DMS1874 via pKD46 mediated recombination. This construct was then P1 transduced into DMS1688 (Δ*rpoS::kan*) to create strain DMS2098.

### DNA microarrays

For all RNA work, strains were inoculated directly from −80°C frozen culture into 1 ml of MOPS MM in a culture tube and grown overnight at 37°C shaken at 200 rpm. The next day, the culture was diluted 1∶100 into MOPS MM and grown for two hours at 37°C, shaken at 200 rpm. NaCl was added to a final concentration of 0.3 M and cultures were grown for another 22 hours. The next day, the culture was diluted 1∶100 into each of two 25 ml volumes of MOPS MM+0.3 M NaCl, and grown until the cells reached an OD_600_ of between 0.25 and 0.3. Growth was stopped by the addition of 5 ml ice-cold phenol∶ethanol (5∶95 by volume), and the cells were left on ice for 20 to 40 minutes. Cells were pelleted by centrifugation, resuspended in Trizol (Sigma-Aldrich) and frozen at −80°C for up to one week before RNA was extracted following the manufacturer's specification. After checking that RNA was not degraded, RNA from both flasks from each of two separate days was pooled. Double-stranded cDNA synthesis followed the instructions of the manufacturer (Invitrogen). Two separate RNA pools were obtained for each strain, resulting in two hybridizations per strain.

cDNA was Cy3 labeled and hybridized to NimbleGen array design 07112, which contains 5 probes per ORF, replicated in two complete blocks. (These were single-color hybridization experiments.) Slides were scanned on a GenePix 4000B scanner and saved as a TIFF file. Data were extracted from the image file and RMA normalized [46] using NimbleScan 2.4 software (NimbleGen). Microarray data are deposited in GEO under accession number GSE13666, and are available as Table S6.

### Transcriptomic data analysis

Log-transformed data were analyzed using the linear modeling approach of Smyth [47] as implemented in the package limma, version 2.16.2 [48] for R, version 2.8.0 [49]. Each array contained two complete block of probes, and these duplicates were used to estimate within array variability as described [50]. A false discovery rate [51] of 0.001 was used as a threshold of distinguishing genes with significant changes of expression. The results of all tests are in Table S7.

### QPCR

Genomic DNA was purified from 50 µl of frozen culture of each line at generation 80, and from strain DMS1745, using the PureGene kit (Gentra). Primer pair P*otsBA* QPCR+ and IS10 out1 were used to specifically amplify the IS*10* insertion into P*_otsBA_*, and primer *rho* QPCR+ and *rho* QPCR− were used to amplify the control gene *rho*. QPCR was performed with the FastStart SYBR Green Master Mix (Roche) on a RotorGene RG-3000 (Corbett Research). Samples were run in duplicate on three separate occasions. The method of Pfaffl [52] was used to quantify the frequency of the P*_otsBA_*::IS*10* in each evolving culture. Purified DNA from strain DMS1745, which contains the P*_otsBA_::*IS*10* insertion, was used the control sample.

### GFP reporter fusions

To measure expression of wild-type P*_otsBA_* and P*_otsBA_*::IS*10*, both promoters were amplified by PCR using primers *otsBA*+NotI and *otsBA*-XbaI and cloned into the promoterless-*gfp* reporter plasmid pZep08 [22]. These plasmids were then transformed [53] into appropriate strains. To measure expression, strains were cultured overnight in MOPS MM, and then diluted 1∶100 into 25 ml fresh MOPS MM and grown for two hours. After two hours, NaCl was added to a final concentration of 0.3 M. This is time = 0 on the plots. Cells were sampled by dilution into 4% formaldehyde in PBS, and stored at 4°C overnight. Fluorescence of 10,000 cells from each sample was measured by flow cytometery.

### Selection of mutants upregulating *otsB::lacZY*

Strain DMS2098 was struck onto L+kanamycin plates, and individual colonies were picked into 1.5 ml volumes of MOPS MM+0.3 M NaCl+kanamycin. Cultures were grown until they reached turbidity, which took 1 to 4 days. The entire volume was spun down, resuspended in MOPS MM lacking glucose or K_2_PO_4_, and spread on MOPS MM plates, with 0.5% lactose instead of glucose, 0.3 M NaCl, kanamycin, and 40 µg ml^−1^ X-gal. Plates were incubated at 37°C for 3 days, after which time a single colony was randomly chosen off each plate and purified on a plate of the same media. Of the 23 isolated mutants, two were excluded because IS*10* was inserted into the CDS of *otsB*. This insertion upregulated *lacZY*, but would knockout *otsB* in a wild-type background.

## Supporting Information

Table S1 Doubling times. Growth rate was measured in MOPS MM+0.3 M NaCl by change in OD_600_. Linear regression of log_2_ transformed measurements between OD_600_ of 0.05 and 1 was used to estimate the doubling time from each of three replicate experiments. R^2^ was greater than 0.99 for all regressions.
(0.03 MB DOC)

Table S2 Genes significantly changed in all 5 evolved Δ*rpoS* lines. The third column gives the change in the Δ*rpoS::kan* relative to wild type, while the fourth gives the average change in the five Δ*rpoS* lines relative to the ancestral Δ*rpoS::kan* line.
(0.03 MB DOC)

Table S3 Frequency of the IS*10* insertion in each Δ*rpoS* culture at generation 80. QPCR was used to measure the frequency of the P*_otsBA_*::IS*10* insertion in each sample. Tukey's HSD test on log-transformed data revealed that all cultures had P*_otsBA_*::IS*10* frequencies significantly different from the all other cultures (p<0.015).
(0.02 MB DOC)

Table S4 Strains used in this study.
(0.10 MB DOC)

Table S5 Primers (5′-3′) used in this study.
(0.05 MB DOC)

Table S6 This file includes the RMA-normalized, log-transformed microarray data used in this study.
(2.89 MB TXT)

Table S7 The results of the FDR adjusted significance tests for all genes of all strains.
(0.23 MB TXT)

Text S1 This file documents the location of IS*10* in the genome of the strain used for experimental evolution.
(0.07 MB PDF)
