# Supplementary material for: Compensatory Evolution of Gene Regulation in Response to Stress by Escherichia coli Lacking RpoS
Source: PLoS Genet. 2009 Oct 2;5(10):e1000671. doi: 10.1371/journal.pgen.1000671 (PMC2744996; doi:10.1371/journal.pgen.1000671)
Supplement: Table S1 — Doubling times. Growth rate was measured in MOPS MM+0.3 M NaCl by change in OD600. Linear regression of log2 transformed measurements between OD600 of 0.05 and 1 was used to estimate the doubling time from each of three replicate experiments. R2 was greater than 0.99 for all regressions. (0.03 MB DOC) [file pgen.1000671.s001.doc]

**Table S1. Doubling times.** Growth rate was measured in MOPS MM + 0.3 M NaCl by change in OD_600_. Linear regression of log_2_ transformed measurements between OD_600_ of 0.05 and 1 was used to estimate the doubling time from each of three replicate experiments. R^2^ was greater than 0.99 for all regressions.

| **Strain** | **Mean doubling time (min) ±**  **standard error of the mean** |
| --- | --- |
| *rpoS^+^* | 77 ± 1.5 |
| *∆rpoS* | 110 ± 8.1 |
| *rpoS*+1 | 72 ± 1.2 |
| *rpoS*+2 | 64 ± 0.1 |
| *rpoS*+3 | 71 ±1.1 |
| *rpoS*+4 | 66 ± 0.9 |
| *rpoS*+5 | 66 ± 0.4 |
| ∆*rpoS*-1 | 79 ± 3.6 |
| ∆*rpoS*-2 | 82 ± 2.1 |
| ∆*rpoS*-3 | 69 ± 1.4 |
| ∆*rpoS*-4 | 82 ± 2.1 |
| ∆*rpoS*-5 | 82 ± 1.1 |
