# Supplementary material for: Compensatory Evolution of Gene Regulation in Response to Stress by Escherichia coli Lacking RpoS
Source: PLoS Genet. 2009 Oct 2;5(10):e1000671. doi: 10.1371/journal.pgen.1000671 (PMC2744996; doi:10.1371/journal.pgen.1000671)
Supplement: Table S2 — Genes significantly changed in all 5 evolved ΔrpoS lines. The third column gives the change in the ΔrpoS::kan relative to wild type, while the fourth gives the average change in the five ΔrpoS lines relative to the ancestral ΔrpoS::kan line. (0.03 MB DOC) [file pgen.1000671.s002.doc]

**Table S2. Genes significantly changed in all 5 evolved ∆*rpoS* lines.** The third column gives the change in the ∆*rpoS*::*kan* relative to wild type, while the fourth gives the average change in the five ∆*rpoS* lines relative to the ancestral ∆*rpoS::kan* line.

| **bNum** | **Gene name** | **Fold change in ∆*rpoS*** | | **Average fold change in five**  **evolved *∆rpoS* lines** |
| --- | --- | --- | --- | --- |
| b0383 | *phoA* | | 3.99 | 0.34 |
| b0953 | *rmf* | | 4.63 | 0.30 |
| b1171 | *ymgD* | | 9.82 | 0.37 |
| b1189 | *dadA* | | 3.23 | 0.33 |
| b1258 | *yciF* | | 0.35 | 0.24 |
| b1304 | *pspA* | | 2.54 | 0.30 |
| b1305 | *pspB* | | 2.43 | 0.33 |
| b1306 | *pspC* | | 2.19 | 0.35 |
| b1453 | *ansP* | | 2.44 | 0.46 |
| b1480 | *sra* | | 3.16 | 0.28 |
| b1599 | *mdtI* | | 4.54 | 0.34 |
| b1675 | *ydhZ* | | 3.45 | 0.27 |
| b1743 | *spy* | | 3.40 | 0.34 |
| b1811 | *yoaH* | | 2.83 | 0.41 |
| b1896 | *otsA* | | 0.07 | 9.67 |
| b1897 | *otsB* | | 0.06 | 12.50 |
| b2149 | *mglA* | | 0.61 | 2.57 |
| b2209 | *eco* | | 4.88 | 0.32 |
| b2939 | *yqgB* | | 0.55 | 2.50 |
| b2965 | *speC* | | 0.43 | 2.34 |
| b3172 | *argG* | | 0.61 | 1.82 |
| b3238 | *yhcN* | | 2.78 | 0.25 |
| b3454 | *livF* | | 0.30 | 3.00 |
| b3455 | *livG* | | 0.27 | 3.06 |
| b3456 | *livM* | | 0.22 | 3.32 |
| b3457 | *livH* | | 0.21 | 2.88 |
| b3458 | *livK* | | 0.27 | 3.37 |
| b3523 | *yhjE* | | 0.37 | 2.76 |
| b3653 | *gltS* | | 2.38 | 0.47 |
| b4035 | *malK* | | 0.22 | 3.70 |
| b4036 | *lamB* | | 0.21 | 3.50 |
| b4037 | *malM* | | 0.19 | 3.87 |
| b4077 | *gltP* | | 2.92 | 0.26 |
| b4111 | *proP* | | 1.86 | 0.42 |
| b4119 | *melA* | | 0.18 | 2.99 |
| b4244 | *pyrI* | | 0.43 | 2.10 |
| b4376 | *osmY* | | 0.19 | 0.38 |
