# Supplementary material for: Compensatory Evolution of Gene Regulation in Response to Stress by Escherichia coli Lacking RpoS
Source: PLoS Genet. 2009 Oct 2;5(10):e1000671. doi: 10.1371/journal.pgen.1000671 (PMC2744996; doi:10.1371/journal.pgen.1000671)
Supplement: Table S3 — Frequency of the IS10 insertion in each ΔrpoS culture at generation 80. QPCR was used to measure the frequency of the PotsBA::IS10 insertion in each sample. Tukey's HSD test on log-transformed data revealed that all cultures had PotsBA::IS10 frequencies significantly different from the all other cultures (p<0.015). (0.02 MB DOC) [file pgen.1000671.s003.doc]

**Table S3. Frequency of the IS*10* insertion in each ∆*rpoS* culture at generation 80.** QPCR was used to measure the frequency of the P*_otsBA_*::IS*10* insertion in each sample. Tukey's HSD test on log-transformed data revealed that all cultures had P*_otsBA_*::IS*10* frequencies significantly different from the all other cultures (p < 0.015).

| Culture | Mean freq ± SEM of P*_otsBA_*::IS*10* |
| --- | --- |
| ∆*rpoS*+1 | 1.69 x 10^-3^ ± 1.17 x 10^-4^ |
| ∆*rpoS*+2 | 5.95 x 10^-5^ ± 1.43 x 10^-6^ |
| ∆*rpoS*+3 | 4.75 x 10^-4^ ± 3.18 x 10^-5^ |
| ∆*rpoS*+4 | 2.84 x 10^-5^ ± 5.10 x 10^-6^ |
| ∆*rpoS*+5 | 2.72 x 10^-4^ ± 1.73 x 10^-5^ |
