# Supplementary material for: Compensatory Evolution of Gene Regulation in Response to Stress by Escherichia coli Lacking RpoS
Source: PLoS Genet. 2009 Oct 2;5(10):e1000671. doi: 10.1371/journal.pgen.1000671 (PMC2744996; doi:10.1371/journal.pgen.1000671)
Supplement: Table S4 — Strains used in this study. (0.10 MB DOC) [file pgen.1000671.s004.doc]

**Table S4: Strains used in this study**

| **strain** | **genotype** | **source** | **citation** |
| --- | --- | --- | --- |
| CF1684 | MG1655, wt | Mike Cashel | [1] |
| CF7968 | MG1655 *rph^+^, ∆lacIZ* | Mike Cashel | [2] |
| ZK1000 | W3110, ∆*rpoS::kan* | Lab collection | [3] |
| JW4081-1 | *∆(araD-araB)567, ∆lacZ4787(::rrnB-3), rph-1, ∆(rhaD-rhaB)568, hsdR514, ∆(melB746::kan)* | CGSC | [4] |
| ES1481 | *lacZ53*(Am), *mutS215*::Tn*10*, *thyA36*, IN(*rrnD*-*rrnE*)1, *rha*-5, *metB1*, *deoC2*, *pur-* | CGSC | [5] |
| DMS1684 | Isolate of CF7968, base strain for experimental evolution |  | This study |
| DMS1688 | DMS1684 ∆*rpoS::kan* from ZK1000 |  | This study |
| DMS1692 | DMS1684 *fhuA* |  | This study |
| DMS1711 | Isolate of CF7986 |  | This study |
| DMS1717 | DMS1711 ∆*rpoS::kan* |  | This study |
| DMS1725 | DMS1684 ∆*rpoS::kan* *fhuA* |  | This study |
| DMS1726 | Isolate of CF7986 |  | This study |
| DMS1727 | DMS1726 ∆*rpoS::kan* |  | This study |
| DMS1735 | Isolated from population *rpoS*+1 after 250 generations of evolution |  | This study |
| DMS1737 | Isolated from population *rpoS*+2 after 250 generations of evolution |  | This study |
| DMS1739 | Isolated from population *rpoS*+3 after 250 generations of evolution |  | This study |
| DMS1741 | Isolated from population *rpoS*+4 after 250 generations of evolution |  | This study |
| DMS1743 | Isolated from population *rpoS*+5 after 250 generations of evolution |  | This study |
| DMS1745 | Isolated from population ∆*rpoS*-1 after 250 generations of evolution |  | This study |
| DMS1747 | Isolated from population ∆*rpoS*-2 after 250 generations of evolution |  | This study |
| DMS1749 | Isolated from population ∆*rpoS*-3 after 250 generations of evolution |  | This study |
| DMS1751 | Isolated from population ∆*rpoS*-4 after 250 generations of evolution |  | This study |
| DMS1753 | Isolated from population ∆*rpoS*-5 after 250 generations of evolution |  | This study |
| DMS1766 | DMS1684 ∆*melB::kan* |  | This study |
| DMS1782 | DMS1737 ∆*rpoS::kan* |  | This study |
| DMS1784 | DMS1739 ∆*rpoS::kan* |  | This study |
| DMS1786 | DMS1741 ∆*rpoS::kan* |  | This study |
| DMS1788 | DMS1743 ∆*rpoS::kan* |  | This study |
| DMS1790 | DMS1745 *ygbM::tetRA* *rpoS^+^* |  | This study |
| DMS1792 | DMS1747 *ygbM::tetRA* *rpoS*^+^ |  | This study |
| DMS1794 | DMS1749 *ygbM::tetRA* *rpoS*^+^ |  | This study |
| DMS1796 | DMS1751 *ygbM::tetRA* *rpoS*^+^ |  | This study |
| DMS1798 | DMS1753 *ygbM::tetRA* *rpoS*^+^ |  | This study |
| DMS1815 | DMS1684 *ygbM::tetRA* *rpoS^+^* |  | This study |
| DMS1874 | CF1684 *araH::tetRA* |  | This study |
| DMS1945 | CF1684 *araH::tetRA* P_otsBA_::IS*10* |  | This study |
| DMS1904 | DMS1684 ∆*rpoS::kan* *araH::tetRA* |  | This study |
| DMS1955 | DMS1684 *∆rpoS::kan* *araH::tetRA* P*_otsBA_*::IS*10* |  | This study |
| DMS1956 | DMS1745 *araH::tetRA* wt PotsBA |  | This study |
| DMS1957 | DMS1747 *araH::tetRA* wt PotsBA |  | This study |
| DMS1958 | DMS1749 *araH::tetRA* wt PotsBA |  | This study |
| DMS1959 | DMS1751 *araH::tetRA* wt PotsBA |  | This study |
| DMS1960 | DMS1753 *araH::tetRA* wt PotsBA |  | This study |
| DMS1961 | DMS1684 ∆*rpoS::kan* *araH::tetRA* *fhuA* |  | This study |
| DMS1962 | DMS1684 *∆rpoS::kan* *araH::tetRA* P*_otsBA_*::IS*10* *fhuA* |  | This study |
| DMS1963 | DMS1745 *araH::tetRA* wt PotsBA *fhuA* |  | This study |
| DMS1964 | DMS1747 *araH::tetRA* wt PotsBA *fhuA* |  | This study |
| DMS1965 | DMS1749 *araH::tetRA* wt PotsBA *fhuA* |  | This study |
| DMS1966 | DMS1751 *araH::tetRA* wt PotsBA *fhuA* |  | This study |
| DMS1967 | DMS1753 *araH::tetRA* wt PotsBA *fhuA* |  | This study |
| DMS2098 | DMS1684 ∆*rpoS::kan araH::tetRA otsB::lacZYcat* |  | This study |
| DMS2239 | MG1655 *mutS215*::Tn*10* from ES1481 |  | This study |
| **Plasmids** |  |  | This study |
| pZep08 | Promoterless gfp reporter plasmid | Lab collection | [6] |
| pDMS123 | pZep08 with wild-type P*_otsBA_* |  | This study |
| pDMS125 | pZep08 with P*_otsBA_*::IS*10* |  | This study |

1. Xiao H, Kalman M, Ikehara K, Zemel S, Glaser G, et al. (1991) Residual guanosine 3',5'-bispyrophosphate synthetic activity of relA null mutants can be eliminated by spoT null mutations. J Biol Chem 266: 5980-5990.

2. Brown L, Gentry D, Elliott T, Cashel M (2002) DksA affects ppGpp induction of RpoS at a translational level. J Bacteriol 184: 4455-4465.

3. Bohannon DE, Connell N, Keener J, Tormo A, Espinosa-Urgel M, et al. (1991) Stationary-phase-inducible "gearbox" promoters: differential effects of katF mutations and role of sigma 70. J Bacteriol 173: 4482-4492.

4. Baba T, Ara T, Hasegawa M, Takai Y, Okumura Y, et al. (2006) Construction of Escherichia coli K-12 in-frame, single-gene knockout mutants: the Keio collection. Mol Syst Biol 2: 2006 0008.

5. Siegel EC, Wain SL, Meltzer SF, Binion ML, Steinberg JL (1982) Mutator mutations in Escherichia coli induced by the insertion of phage mu and the transposable resistance elements Tn5 and Tn10. Mutat Res 93: 25-33.

6. Hautefort I, Proenca MJ, Hinton JC (2003) Single-copy green fluorescent protein gene fusions allow accurate measurement of Salmonella gene expression in vitro and during infection of mammalian cells. Appl Environ Microbiol 69: 7480-7491.
