# Supplementary material for: Compensatory Evolution of Gene Regulation in Response to Stress by Escherichia coli Lacking RpoS
Source: PLoS Genet. 2009 Oct 2;5(10):e1000671. doi: 10.1371/journal.pgen.1000671 (PMC2744996; doi:10.1371/journal.pgen.1000671)
Supplement: Table S5 — Primers (5′-3′) used in this study. (0.05 MB DOC) [file pgen.1000671.s005.doc]

**Table S5** **Primers (5'-3') used in this study:**

| **For *ygbM*** |  |
| --- | --- |
| *ygbM*_tetRA+ | CTGACCGCAGGTGAAATTGCGGAATTAAGGAGTTAATGCAttaagacccactttcacatt |
| *ygbM*_tetRA- | GGATGTATGGGTACGTTGTAATTAGGGATTTAACGAATTActaagcacttgtctcctg |
| *ygbM*_sequence+ | GAACTGGCAGCAGACAATCA |
| *ygbM*_sequence- | CAGCATGATTACGCCAGCTA |
|  |  |
| **For *araH*** |  |
| *araH*_tetRA+ | ctgcttaatatttctcctttcgcgcagtacgtggttcgcTTAAGACCCACTTTCACATT |
| *araH*_tetRA- | tgtaacggtcgaagatcaccgctgccagcaggattaagccCTAAGCACTTGTCTCCTG |
| *otsB*_recomb- | CAAAAAACCAGGCATATTTCGCGGATAGTTCAGGGGTTTC |
| *araH*_*tetRA* verify+ | TATGTGGTGGCGGGTATCTT |
| *araH*_*tetRA* verify- | ACGGGAAGCGATAAGGTTTT |
|  |  |
| **For QPCR** |  |
| P*otsBA*_QPCR+ | CCATAACGGTTGGCTGTTCT |
| IS*10*_out1 | AACAAGGTTGGGACAAGCAC |
| *rho*_QPCR+ | AACCTCCGCACTGGTGATAC |
| *rho*_QPCR- | AGATCCAGTACGCGAGCAGT |
| IS*10*_QPCR2+ | GGCATGAGATTGGATTGCTT |
| IS*10*_QPCR2- | TAGGAGCGGAAAACTGGAAA |
|  |  |
| **For *otsB::lacZYcat* fusion** |  |
| *cat*_*lacA*+ | TTGAACATGCCAATGACCGAAAGAATAAGAGCAGGCAAGCGTGTAGGCTGGAGCTGCTTC |
| *cat*_*lacA*- | AGTCTTTTTTCCGGTAAGCCTTCGCACATATCGGTAAATACATATGAATATCCTCCTTAG |
| *otsB*_cds_*lacZ*_fusion+ | ATTGCGCGTGATATTAGCGTGCAACTGCATACAGTCTAATGAGCGGATAACAATTTCACA |
| otsA_cds_*lacZ*_fusion- | CAAATTGCGAAAGAACAAGAACGCCCGGATTGGCTGGGTCCATATGAATATCCTCCTTAG |
| *otsB*_cds+ | CAGAACCGTTAACCGAAACC |
| *lacZ*_*otsB*_cds- | GTTTTCCCAGTCACGACGTT |
|  |  |
| **For *otsBA* *gfp* fusion** |  |
| *otsBA*+NotI | aacgcggccgcTATTTCTCCTTTCGCGCAGT |
| *otsBA*-XbaI | cgctctagaACGGGAAGCGATAAGGTTTT |
