# Supplementary material for: Compensatory Evolution of Gene Regulation in Response to Stress by Escherichia coli Lacking RpoS
Source: PLoS Genet. 2009 Oct 2;5(10):e1000671. doi: 10.1371/journal.pgen.1000671 (PMC2744996; doi:10.1371/journal.pgen.1000671)
Supplement: Text S1 — This file documents the location of IS10 in the genome of the strain used for experimental evolution. (0.07 MB PDF) [file pgen.1000671.s008.pdf]

Supporting text 1:
The location of IS*10*

The copy number and location of IS*10 in* CF7968, the parental strain for all of the experiments in this paper, was unknown. To determine the number and location of these elements in the genome of our strain, we employed vectorette PCR, which has previously been demonstrated to be a reliable technique for mapping IS elements in the genome of *E. coli* [1]. Using the modified primers of [2], IS*10* was discovered in two locations of the genome of DMS1688, and confirmed by amplification and sequencing with flanking primers. The two locations are:

1) **Upstream of *hlyE*.** IS*10* is inserted 144bp upstream of the start codon of *hlyE*, with P_{OUT} oriented away from *hlyE*.

2) **At the very end of *ydiM***. IS*10* is inserted 7bp from the end of *ydiM*, resulting in the stop codon occurring one amino acid earlier than wild-type. P_{OUT} is oriented towards the downstream gene, *ydiN*.

We used QPCR to measure the number of copies of IS*10* in the genome of DMS1688, using *rho* as a control gene. Primers IS*10*_QPCR2+ and IS*10*_QPCR2- were used to measure IS*10* copy number, and primers *rho*_QPCR+ and *rho*_QPCR- were used for *rho* (Table S5). The control strain, DMS2239, was a MG1655 derivative into which *mutS215::*Tn*10* had been introduced by P1 transduction.

Using the method of Pfaffl [3], the estimated number of copies of IS*10* in DMS1688 was 1.75. With a 95% confidence interval of 1.22 to 2.27 copies, these results are consistent with our strain containing only two copies of IS*10*.

Literature cited:

1. Zhong S, Dean AM (2004) Rapid identification and mapping of insertion sequences in Escherichia coli genomes using vectorette PCR. BMC Microbiol 4: 26.
2. Ko WY, David RM, Akashi H (2003) Molecular phylogeny of the Drosophila melanogaster species subgroup. J Mol Evol 57: 562-573.
3. Pfaffl MW (2001) A new mathematical model for relative quantification in real-time RT-PCR. Nucleic Acids Res 29: e45.
